# Drug Treatment of Patients with Liver Cirrhosis in a Tertiary Hospital in Northern Ghana: Does It Comply with Recommended Guidelines?

**DOI:** 10.1155/2020/9750194

**Published:** 2020-05-28

**Authors:** Baba Sulemana Mohammed, Matthew Aidoo

**Affiliations:** ^1^Department of Pharmacy, University for Development Studies, Tamale, Ghana; ^2^Tamale Teaching Hospital, Tamale, Ghana

## Abstract

The diverse influence of liver function on drug disposition can lead health-care practitioners to inappropriate drug selection, inappropriate drug dosing, or some level of therapeutic negativism. The aim of this study was to assess how drug prescribing in patients with liver cirrhosis at the Tamale Teaching Hospital comply with recommendations of pharmacotherapy and safety guidelines. A prospective cross-sectional study was conducted from February to July, 2019, at the medical ward of the Tamale Teaching Hospital. A total of 152 liver cirrhotic patients were included in this study. Common etiologies for liver cirrhosis were chronic hepatitis B 80 (52.6%) and chronic hepatitis C 30 (19.7%); about 12.5% of etiologies were unknown. Of the 1842 prescription issued, 69% (1270/1842) were compliant. Of the 572 noncompliant prescriptions, about 32% (183/572) were due to pharmacotherapy and 68% (389/572) due to safety guideline recommendations. There was a substantial number (31%) of prescription noncompliance with recommendations for pharmacotherapy and safety guidelines in liver cirrhotic patients at the tertiary hospital in northern Ghana. Prescribers need to be conscious of the role of the liver in drug elimination and prescribe as recommended by guidelines.

## 1. Introduction

Liver cirrhosis is one of the complications of chronic liver diseases (CLDs), and the pathophysiology which occurs in liver cirrhosis has the potential to alter pharmacokinetics and pharmacodynamics [1]. These changes generally can result in higher drug levels and possibly cause unwanted side effects and toxicity in patients with liver cirrhosis [2].

Prescribing drugs in patients with liver cirrhosis is challenging because of concerns that the drug may exacerbate the liver disease. There is also the fear that the altered liver state may change metabolism and excretion of the drug [3]. About 50% of drugs have been associated with liver injury, and more than 100 drugs are implicated in fulminant hepatic failure, and 10% of all adverse drug reactions are hepatotoxic effects [4].

Patients with CLDs require appropriate drug therapy for the etiology and also the associated complications, including cirrhosis of the liver. Drug formulary references give recommendations on drugs that should be used with caution or avoided, and when unavoidable, their dosage be adjusted in patients with CLDs [5]. The World Health Organization (WHO), European Association for the Study of Liver (EASL), and American Association for Study of Liver Disease (AASLD) among others provide guidelines that have been formulated from evidence-based practice for the management and treatment of the etiology and complications of liver disease. A review of literature, however, indicates that there is no data on drug utilization review among CLD patients in Ghana. The aim of this study was therefore to assess the compliance of pharmacotherapy in patients with liver cirrhosis at the Tamale Teaching Hospital with evidence-based guidelines and drug formulary recommendations.

## 2. Materials and Methods

### 2.1. Study Design and Site

A cross-sectional prospective study was conducted involving patients diagnosed with cirrhosis at the medical ward of the Tamale Teaching Hospital (TTH). The TTH is a tertiary and referral hospital for the northern sector of Ghana and also an institution for training of health professionals. With a bed capacity of 450, the TTH sees over 100,000 patients a year. The medical ward is run by the internal medicine department and has a bed capacity of 216. At the time of the study, there were 5 physician specialists, 6 medical officers, and 24 house officers manning the medical ward. There were also two specialist pharmacists and 6 pharmacists at the ward. At the TTH, it is the sole role of the doctor to diagnose and prescribe treatment for the patient. The pharmacist is responsible for drug information, procurement, storage, and dispensing of pharmaceuticals to the patient in accordance with the prescription of the doctor. Clinical consultation between the doctor and the pharmacist is not formalized. There is no electronic prescribing platform, and prescribing is supported largely by the clinical judgment of the doctor.

### 2.2. Patients and Inclusion Criteria

Patients admitted at the medical ward of the TTH between February and July, 2019, and diagnosed with a chronic liver disease were eligible for the study. Patients were only included in the study if they were ≥18 years of age and had liver cirrhosis. The criteria for diagnosis of chronic liver disease were that patients must have a clinical history of liver disease (elevated liver enzymes, high bilirubin, and/or low albumin levels) over a period of at least 6 months. Liver biochemical values were obtained from the hospital's laboratory reports of liver function test and compared with reference values (Appendix 1). Diagnosis of hepatitis B was made by laboratory confirmation of positive hepatitis B surface antigen (HBsAg) for at least six months (Appendix 2).

The criteria for diagnosis of liver cirrhosis included (1) confirmatory diagnosis with abdominal imaging (e.g., ultrasound, computed tomography, or magnetic resonance imaging) showing nodular liver surface and a coarse echo pattern in the liver parenchyma with enlargement or shrinkage of the liver, splenomegaly, and/or ascites [6] or (2) clinical diagnosis—using clinical features of decompensated cirrhosis phase, including ascites, bleeding, jaundice, or hepatic encephalopathy [6, 7].

### 2.3. Data Collection

Data was collected from the patient's medical records, using a specially designed form. Patient's demographic data included sex and age. Clinical data collected were etiology and severity of the liver cirrhosis and other complications of chronic liver disease. Diagnosis of the etiology and complications of liver cirrhosis was made according to the criteria in Appendix 2 and Appendix 3, respectively. Severity of the liver cirrhosis was assessed according to the Child Pugh's classification. Data on drug treatment consisted of all medications prescribed for etiology and complications of liver cirrhosis.

### 2.4. Assessment of Compliance with Guidelines

Compliance with pharmacotherapy was assessed in two categories: according to recommendation for prescribing first choice therapy and recommendation for safe prescribing.

In assessing compliance, according to first choice therapy, guidelines from World Health Organization (WHO), American Association for Study of Liver Disease (ASSLD), and European Association for the Study of the Liver (EASL) shown in Appendix 4 and Appendix 5 were used.

With respect to safe prescribing, the safety recommendations by Weersink et al. [8] (Appendix 6) and dosing considerations in liver impairment by the British National Formulary and/or Medscape were utilized (Appendix 7).

### 2.5. Data Analysis

Descriptive statistics of the patients' demographic and clinical characteristics were performed and summarized in percentages and presented in tables. Statistical Package for Social Sciences (SPSS), version 25 (IBM. Illinois, USA), was used for the data analysis.

### 2.6. Ethical Considerations

Authorization to conduct the research was sought from the management of the TTH through an application containing a summary of the study proposal and a copy of the data collection tool. Approval was granted before the study commenced.

## 3. Results

### 3.1. Characteristics of Patients

A total of 152 patients with liver cirrhosis who met the inclusion criteria were involved in the study, with majority (71.7%) being males (Table 1). The mean (s.d) age of the patients was 41 (13.20) years, and most (43.4%) of them were in the 18-39-year group. With respect to severity of the disease, almost half (49%; 74/152) of the patients were of Class B (moderate cirrhosis), with a little above 10% (16/152) being in Class A (mild cirrhosis). Hepatitis B virus accounted for majority 80 (52.6%) of the etiology of the liver cirrhosis. Alcohol was the cause in only 8 (5.3%) of the cases. The etiology was not known (cryptogenic) for 19 (12.5%) of the patients (Table 1). Other common complications were ascites, which was seen in 120 (78.9%) of patients, followed by hypoalbuminemia in 111 (73.0%). Jaundice and spontaneous bacterial peritonitis (SBP) were also seen in majority of the patients and were present in 86 (56.6%) and 85 (55.9%) of patients, respectively (Table 1). Gastroesophageal varices formed the least common complication and was present in 13 (8.6%) of the study participants.

### 3.2. Medicines Prescribed to Patients

There were 1842 prescription episodes comprising 39 medicines written for the 152 patients studied; at least 46 (2.5%) prescriptions were for etiologies and 1796 (97.5%) for complications. For the 133 patients with known etiologies, 34.6% (46/133) were issued prescriptions (Table 2). For the 80 hepatitis B viral cause, 23 (28.8%) were prescribed an antiviral; 21 (26.3%) prescribed tenofovir and 2 (2.5%) lamivudine. Only four (13.3%) of the 30 hepatitis C viral causes were prescribed an antiviral medicine; three (10.0%) were given a combination of sofosbuvir/ledipasvir, while one patient was put on ribavirin. Five (33.3%) of the 15 patients with HCC were given sorafenib. Thiamine was prescribed to 7 (87.5%) patients with alcohol as a cause of their liver cirrhosis.

The top 10 therapeutic classes of medicines utilized for the management of complications of liver cirrhosis are presented in (Table 3).

A total of 1796 medicines were prescribed, with antibacterial agents being the most utilized class at 19.2% (345/1796) and beta-blockers being the least at 3.7% (66/1796). Among the antibacterial agents, metronidazole was the most prescribed with 45.2% (156/345) prescriptions written for 119 patients. Furosemide made up the majority of the diuretic prescriptions, with 58% (196/338) made to 126 patients. Glucose powder at 41% (89/217) was the most utilized among the glucose-elevating agents and was written for 89 of the 152 patients, while tramadol with 58% (114/197) was the major analgesic prescribed to 103 patients. Among the vitamin supplements, vitamin B complex at 35.2% (69/196) was the most, with 69 prescriptions made to 29% (44/152) of the patients. The main prescribed medicine among the anti-acid-secreting agents was omeprazole with 114 prescriptions made to about 94 of patients.

Among the 90 hematinics, folic acid, 34 (37.8%), and ferrous sulphate, 33 (36.7%), constituted the main prescribed agents. Prescription pattern of blood products indicated albumin at almost 37% (30/82) to be the highest prescribed. Propranolol was the only agent recommended under the class of beta-blockers.

### 3.3. Compliance with Guideline Recommendations

Of the 1842 prescription issued, 69% (1270/1842) were compliant. Of the 572 noncompliant prescriptions, about 32% (183/572) were due to first choice therapy and 68% (389/572) due to safety guidelines recommendations. With regard to first choice therapy, about 10% (183/1842) of all prescription written was noncompliant. Metronidazole was the highest in this category, with 98 prescriptions made to 55.9% (85/152) of patients, whereas there was only one prescription for ribavirin (Table 4).

According to safe prescribing guideline recommendations, a total of 389 (21.1%) of the total prescriptions were not compliant (Table 5). There were 23.7% (36/152) of patients who received at least one prescription of omeprazole, which was unsafe. A total of 99 prescriptions of omeprazole higher than the maximum dose were made to 51.3% (78/152) of patients. Tramadol was the lowest noncompliant medicine with respect to safety, where 32 prescriptions of it were made in higher than the recommended frequency to 17.1% (26/152) of patients (Table 5).

## 4. Discussion

Hepatitis B virus was the major cause of liver cirrhosis, and this agrees with other reports from Africa and Asia [9–12]. Drug utilization for etiology of liver cirrhosis revealed that tenofovir was the main antiviral agent for treatment of CHB. Tenofovir may have been preferred because it is highly potent, it confers a good barrier to the development of resistance from the HBV, and it is relatively inexpensive [13]. Antibacterial agents were the most frequently prescribed therapeutic class of drugs for complications of liver cirrhosis, and this was consistent with findings reported by other studies [11, 14].

Analysis of drug prescribing compliance with recommendations of pharmacotherapy guidelines revealed several instances of noncompliance. For instance, two (1.3%) patients with CHB infection had lamivudine prescribed in a noncompliant manner according to first-line therapy indication for CHB. The guideline-recommended therapy for CHB is drugs of the nucleoside/nucleotide analogues, such as tenofovir and entecavir that have high barrier to drug-resistant HBV [13, 15, 16]. Lamivudine has low genetic barrier to drug-resistant HBV, and many patients worldwide have developed resistance to its use [17]. Sofosbuvir/ledipasvir was prescribed for 3 (2.0%) patients with CHC without identification of the HCV genotype, which does not conform with guideline recommendations. This is because sofosbuvir/ledipasvir is a genotypic direct acting antiviral agent indicated for CHC patients with genotypes 1, 4, 5, and 6 [18]. Such prescribing could result in ineffective therapy, especially because HCV genotype 2 has been identified as the most common in Ghana [19].

It was observed that patients with hepatitis B and C infections were mostly not prescribed any treatment. The reason for this observation is not clear to us, but the absence of base-line quantitative HBV DNA data before initiation of antiviral therapy could be blamed in the case of patients with CHB. This data was mostly unavailable because patients may not have been able to pay for the cost. A similar reason has been reported elsewhere, where 69% of patients with no HBV DNA testing did not receive treatment for HBV [20]. However, the WHO recommends treatment of all patients with CHB and clinical evidence of compensated or decompensated cirrhosis regardless of ALT levels, HBeAg status, or HBV DNA levels [13]. Since the patients had clinical and/or confirmed evidence of cirrhosis, they were eligible for treatment. The reason for patients with CHC not receiving treatment may also be patients' inability to afford the high cost of the HCV RNA test and antivirals for HCV.

Evaluation of treatment of complications revealed considerable nonconformity with guideline recommendations. There was widespread prescription of metronidazole for treatment and prophylaxis of spontaneous bacterial peritonitis (SBP). Metronidazole has typical anaerobic antibacterial spectrum and a relatively little effect against aerobic bacteria and as such is not recommended for SBP. The reason is that anaerobic organisms are rare in SBP because of the high oxygen tension of ascitic fluid [21]. Typically, SBP is caused by aerobic organisms, about 75% by aerobic gram-negative organisms and the remaining 25% due to aerobic gram-positive organisms [22]. As such, third-generation cephalosporins and fluoroquinolones, such as ciprofloxacin, are recommended for empirical treatment and prophylaxis of SBP [23, 24]. Also, there was inappropriate prescription of omeprazole (a proton pump inhibitor) for cirrhotic patients with symptoms of upper gastrointestinal bleeding, where endoscopic confirmation of peptic ulceration was not done. In this case, the treatment of gastrointestinal bleeding could be ineffective. It has been reported that the prevalence of peptic pathology in cirrhotic patients is not more than 20%; however, over 60% of patients with liver cirrhosis are prescribed proton pump inhibitors [25]. In cirrhotic patients who present with acute upper gastrointestinal bleeding, variceal hemorrhage must be suspected and treatment initiated with vasoactive drugs (e.g., somatosatin, terlipressin, or octreotide) and antibiotic prophylaxis [7, 26].

In the current study, there was noncompliance with guidelines on safety and dosing recommendations. Almost 24% of patients with Child-Turcotte-Pugh (CTP) C were unsafely prescribed omeprazole. Omeprazole is categorized as “unsafe” in patients with CTP C (severe liver cirrhosis) due to the significant alterations of its pharmacokinetics, which recommends that it is avoided in these patients [27]. Again, omeprazole is recommended to be used at a maximum dose of 20 mg/day in CTP A and B, as it does not increase harm in these patients [26]. It was however found in the current study that there was the prescribing of omeprazole above 20 mg/day to 65% patients with liver cirrhosis (CTP A+B+C).

Metronidazole is recommended to be prescribed at a dose of one-third of the total daily dose 24 hourly [28] or at 50% dose reduction in patients with severe hepatic impairment (CTP C). This downward adjustment of dose is essential to match the reduced metabolism of metronidazole, which has been shown to increase its elimination half-life with manifestation of adverse effects in severe hepatic impairment [29]. In more than 50% of the patients with severe hepatic impairment (CTP C) studied, the prescriptions of metronidazole were not compliant with guideline recommendation of dose reduction.

The combination of spironolactone and furosemide is recommended in patients with long standing and recurrent ascites because it reduces the time to accomplish natriuresis. A dose ratio of 100 mg spironolactone to 40 mg furosemide (maximum dose of 400 mg spironolactone to 160 mg furosemide) is recommended by guidelines [7] as the ratio helps to maintain normokalemia. In this study, 59% of the patients were prescribed spironolactone/furosemide combination that had higher amounts of frusemide, which reduced the ratio to twice or thrice the recommended. The hypokalemia that may result from this could precipitate or worsen hepatic encephalopathy.

It is recommended to limit therapy of paracetamol to short-term use at doses not exceeding 2 g/day in patients with hepatic impairment [30]. In this study, it was noted that, about 32% of patients with severe hepatic impairment (CTP C) received prescriptions of paracetamol exceeding the recommended daily dose. This is a worrying observation for an already compromised liver as the metabolism of paracetamol is extensively hepatic. It is important to mention that nonsteroidal anti-inflammatory drugs (NSAIDs) were not prescribed in this setting unlike in the Netherlands where NSAIDs were among the five most commonly used drugs [8]. In patients with severe hepatic impairment (CTP C), immediate-release tramadol at a dose of 50 mg every 12 hours is recommended [30]. Analysis of tramadol prescription revealed that 17% of patients with CTP C had their dose frequency higher than recommended. Opioids are generally recommended to be used cautiously and initiated with intermediate-release formulations at low doses with extended frequency of administration in patients with liver impairment because of potential accumulation [31].

The level of noncompliance with guideline recommendations in the treatment of patients with liver cirrhosis as we have observed is a reflection of the lack of formal consultation between doctors and pharmacists in the care of patients in Ghana. It is important that both doctors and pharmacist appreciated the harm that medication errors can cause to patients, which requires that these health personnel work together to reduce the harm. Weersink and co-workers [8] provide a useful resource that can guide all health personnel to attain optimal pharmacotherapeutic management of patients with liver diseases. It has recently been demonstrated that pharmacist interventions were able to resolve close to 60% of medication-related problem in patients with decompensated liver cirrhosis [32].

It was remarkable to observe that with regards to patients with HBC viral-associated cirrhosis, only 10% were prescribed an antiviral, albeit in a noncompliant manner. This, together with many of the issues of noncompliance with respect to the choice of treatment and safety of medicines used, means that patients with liver cirrhosis at the TTH are not treated well.

From our search in the literature, this is the first report on prescribing to patients with liver cirrhosis in Africa, which therefore makes it difficult to compare the practice in TTH with others elsewhere in Africa. This is not surprising since liver cirrhosis has been considered a neglected condition in Africa, in particular sub-Saharan Africa. As such, there is limited research on the topic, including prescribing data [33].

## 5. Conclusion

Our study revealed that most drug prescribing in patients with liver cirrhosis was not compliant with pharmacotherapy and safe prescribing recommendations as outlined in guidelines. It is observed that generally no attempt was made by prescribers to adjust downwardly the doses of drugs used in liver cirrhotic patients, and this could expose patients to the risk of drug accumulation and potential adverse drug effects. Health care practitioners working in the northern part of the country need to embrace evidence-based medicine and practice within guideline recommendations to reduce the burden of chronic liver diseases that is common in Ghana.

## Figures and Tables

**Table 1:** Characteristic of patients involved in the study (*n* = 152).

Characteristics	Frequency	Percentage
Sex		
Male	109	71.7
Female	43	28.3
Age range (years)		
18–39	66	43.4
40–59	63	41.4
≥60	23	15.1
Severity		
Class A	16	10.5
Class B	74	48.7
Class C	62	40.8
Etiology		
Hepatitis B	80	52.6
Hepatitis C	30	19.7
Hepatocellular carcinoma	15	9.9
Alcoholic hepatitis	8	5.3
Cryptogenic	19	12.5
Complications		
Ascites	120	78.9
Hypoalbuminemia	111	73.0
Jaundice	86	56.6
Spont. bact. peritonitis	85	55.9
Gastrointestinal bleeding	60	39.5
Portal hypertension	36	23.7
Hyponatremia	26	17.1
Acute kidney injury	20	13.2
Hepatic encephalopathy	18	11.8
Varices	13	8.6

**Table 2:** Medicines prescribed for etiologies of liver cirrhosis (*n* = 133).

Etiology	*N*	Medicines utilized	Dose range	*P* (%)
CHB	80	Tenofovir	300 mg od	21 (26.3)
		Lamivudine	150 mg od	2 (2.5)
CHC	30	Sofosbuvir/ledipasvir	400/90 mg od	3 (10.0)
		Ribavirin	400 mg bd	1 (3.3)
HCC	15	Sorafenib	400 mg bd	5 (33.3)
ALD	8	Thiamine	100 mg od	7 (87.5)
		Baclofen	10 mg tds	4 (50.0)
		Diazepam	10 mg bd/tds	3 (37.5)

*n*: total number of patients with known etiology; *N*: number of patients for each etiology; *P*: number of patients for each etiology treated; CHB: chronic hepatitis B; CHC: chronic hepatitis C, ALD: alcoholic liver disease; HCC: hepatocellular carcinoma; od: every 24 hourly; bd: 12 hourly; tds: 8 hourly.

**Table 3:** Top ten classes of medicines prescribed for patients with liver cirrhosis (*n* = 1796).

Therapeutic class	Dose range	*N* (%)	NP (%)
**Antibacterial agents**		**345 (19.2)**	
Metronidazole	400–500 mg tds	156 (45.2)	119 (78.3)
Ciprofloxacin	400–500 mg bd	97 (28.1)	77 (50.7)
Ceftriaxone	1–2 g od/bd	92 (26.7)	80 (52.6)
**Diuretics**		**338 (18.8)**	
Furosemide	40–80 mg bd/tds	196 (58.0)	126 (82.9)
Spironolactone	50–200 mg od/bd	134 (39.6)	116 (76.3)
Metolazone	5–10 mg od	8 (2.4)	8 (5.3)
**Glucose agents**		**217 (12.1)**	
Glucose powder	100 g qid	89 (41.0)	89 (58.6)
Dextrose 10%	1–2 L/day	50 (23.0)	48 (31.6)
Dex. 5%/saline 0.9%	1–2 L/day	43 (19.8)	42 (27.6)
Dextrose 5%	1–2 L/day	35 (16.1)	34 (22.4)
**Analgesics**		**197 (11.0)**	
Tramadol	50–100 mg bd/tds	114 (57.9)	103 (67.8)
Paracetamol	1 g bd/tds/qid	66 (33.5)	58 (38.2)
Morphine	5–10 mg bd/tds	12 (6.1)	12 (7.9)
Pethidine	50–100 mg bd/tds	5 (2.5)	5 (3.3)
**Vit. supplements**		**196 (10.9)**	
Vitamin B complex	1 tab tds	69 (35.2)	44 (29.0)
Multivitamin	1 tab tds	58 (29.6)	56 (36.8)
Pabrinex	1–2 vial bd/tds	36 (18.4)	36 (23.7)
Vitamin K	10 mg od	19 (9.7)	17 (11.2)
Hepatovit	1 tab od	14 (7.1)	14 (9.2)
**Anti-acid agents**		**134 (7.5)**	
Omeprazole	20–40 mg od/bd	114 (85.1)	94 (61.8)
Antacids	15 ml tds	20 (14.9)	20 (13.2)
**Laxatives**		**131 (7.3)**	
Lactulose	5–15 ml tds	131 (100)	123 (80.9)
**Hematinics**		**90 (5.0)**	
Folic acid	1 tab od	34 (37.8)	30 (19.7)
Ferrous sulphate	1 tab od	33 (36.7)	29 (19.1)
Tothema	1vial od/bd	19 (21.1)	19 (12.5)
Iron dextran	1–2 vial/day	4 (4.4)	4 (2.6)
**Blood products**		**82 (4.6)**	
Albumin	2–3 unit/day	30 (36.6)	30 (19.7)
Packed red cells	2–3 unit/day	22 (26.8)	22 (14.5)
Whole blood	2–3 unit/day	19 (23.2)	19 (12.5)
Fresh frozen plasma	2–3 unit/day	11 (13.4)	11 (7.2)
**Beta-blockers**		**66 (3.7)**	
Propranolol	40 mg od/bd	66 (100)	66 (43.4)

*n*: total number of prescriptions made; *N*: number of prescriptions; NP: number of patients who received prescriptions; od: every 24 hourly; bd: 12 hourly; tds: 8 hourly; qid: 6 hourly.

**Table 4:** Prescriptions not compliant with pharmacotherapy guidelines for indication (*n* = 183).

Drug	Indication	Comment	*N*	NP (%) (*T* = 152)
Metronidazole	SBP	Not first choice	98	85 (55.9)
Omeprazole	GIB	Not first choice	79	54 (35.5)
Sofosbuvir/ledipasvir	CHC	Not first choice	3	3 (2.0)
Lamivudine	CHB	Not first choice	2	2 (1.3)
Ribavirin	CHC	Not first choice	1	1 (0.7)

*n*: total number of prescriptions not compliant according to indication; *N*: number of prescriptions not compliant with guidelines; NP: number of patients who received prescriptions; *T*: total number of patients; CHB: chronic hepatitis B, CHC: chronic hepatitis C, SBP: spontaneous bacterial peritonitis, GIB: gastrointestinal bleeding.

**Table 5:** Prescriptions not compliant with safety prescribing guidelines (*n* = 389).

Drug	Comment	*N*	NP (%) (*T* = 152)
Omeprazole^1^	Avoid		
	Unsafe	36	36 (23.7)
	Dosing consideration		
Omeprazole^2^	Maximum dose exceeded	99	78 (51.3)
Spironolactone+furosemide^3^	Incorrect dose ratio	93	89 (58.6)
Metronidazole^4^	Incorrect dose and frequency	75	64 (42.1)
Paracetamol ^5^	Recommended dose exceeded	54	48 (31.6)
Tramadol^6^	Higher dosage frequency	32	26 (17.1)

*n*: total number of prescriptions not compliant due to safety; *N*: number of prescriptions made; NP: number of patients who received prescriptions; *T*: total number of patients; 1 = omeprazole is classified as “unsafe” in CPT (Child-Turcotte-Pugh) C; 2 = omeprazole maximum dose is 20 mg/day in CTP A, B, and C (hepatic impairment); 3 = spironolactone : furosemide dose ratio is 100 mg : 40 mg to maximum of 400 mg : 160 mg; 4 = metronidazole requires 50% dose reduction in CTP C (severe hepatic impairment); 5 = paracetamol is recommended at a dose of 2 g/day in CTP A, B, and C (hepatic impairment); 6 = tramadol is recommended at a dose of 50 mg 12 hourly in CTP C (severe hepatic impairment).

## Data Availability

The patient and drug data used to support the findings of this study are included within the article.

## Supplementary Materials

Supplementary Materials Appendix 1: criteria for liver function test. Appendix 2: criteria for diagnosis of etiology of liver cirrhosis. Appendix 3: criteria for diagnosis of complications of liver cirrhosis. Appendix 4: guideline recommendation for etiology treatment. Appendix 5: guideline recommendation for complication treatment. Appendix 6: guidelines on safety prescription. Appendix 7: dosing consideration in hepatic impairment.
