# Supplementary material for: Drug Treatment of Patients with Liver Cirrhosis in a Tertiary Hospital in Northern Ghana: Does It Comply with Recommended Guidelines?
Source: Int J Hepatol. 2020 May 28;2020:9750194. doi: 10.1155/2020/9750194 (PMC7275961; doi:10.1155/2020/9750194)
Supplement: Supplementary Materials — Appendix 1: criteria for liver function test. Appendix 2: criteria for diagnosis of etiology of liver cirrhosis. Appendix 3: criteria for diagnosis of complications of liver cirrhosis. Appendix 4: guideline recommendation for etiology treatment. Appendix 5: guideline recommendation for complication treatment. Appendix 6: guidelines on safety prescription. Appendix 7: dosing consideration in hepatic impairment. [file 9750194.f1.pdf]

Appendixes

Appendix 1: Criteria for liver function test

Parameter	Reference range
Total protein	66–83 g/L
Albumin	35–53 g/L
Globulin	20–40 g/L
Aspartate aminotransferase (AST)	0–35 IU/L
Alanine aminotransferase (ALT)	0–45 IU/L
Alkaline phosphatase (ALP)	30–120 IU/L
Gama-glutamyl transpeptidase (GGT)	0–49 U/L
Total bilirubin	2–21 umol/L
Direct bilirubin	≤ 5.13 umol/L
Indirect bilirubin	1.7–17 umol/L

Appendix 2: Criteria for diagnosis of etiology of liver cirrhosis

Etiology of liver cirrhosis	Criteria for diagnosis
Chronic hepatitis B viral infection	• Positive hepatitis B surface antigen (HBsAg) for at least 6 months [11]
Chronic hepatitis C viral infection	• Positive hepatitis C virus (HCV)-RNA for at least 6months [16]
Alcoholic hepatitis	• History of alcohol intake of > 3 standard drinks/day in men and > 2 drinks standard/day in women for ≥ 6 months,<br>• Elevated liver enzymes; AST and ALT > 1.5 times upper limit of normal but < 400 IU, AST/ALT ratio > 1.5<br>• Elevated serum total bilirubin > 3mg/dl (51 umol/L)<br>• In the absence of other causes of liver disease. [34]
Hepatocellular carcinoma	• Abdominal imaging (ultrasound) showing small hypoechoic/hyperechoic nodules or large mixed echogenic nodules together with elevated serum alpha fetoprotein > 400 ng/ml [35]
Cryptogenic liver disease	• A diagnosis was made when no etiology was identified after extensive investigations. [36]

Appendix 3: Criteria for diagnosis of complications of liver cirrhosis

Complication	Criteria for diagnosis
Ascites	• Clinical presence of abdominal distention with fluid thrill and shifting dullness or<br>• Presence of free intraperitoneal fluid by abdominal imaging (e.g. ultrasound) [23]
Coagulopathy	• Clinical presence of spontaneous bleeding or easy bruising and prothrombin time (PT) > 4 secs of the control [37] in the absence of anticoagulation therapy and other causes of coagulopathy.
Gastrointestinal bleeding	• Clinical presence of hematemesis, melena, or hematochezia
Esophageal varices	• Findings of collateral veins/vessels within the walls of the esophagus by imaging techniques e.g. endoscopy. [38]
Hepatic encephalopathy	• Neurologic and psychiatric manifestations, including alterations in intellectual capacity, memory, emotional, behavioral, psychomotor speed, and fine motor skills. [39].
Jaundice	• Clinical presence of yellowish coloration of the sclera, and/or skin.
Portal hypertension	• Clinical presence of distended superficial abdominal veins (caput medusa) and/or<br>• Findings of dilated portal vein, collateral vessels/varices, splenomegaly, and ascites by imaging studies e.g. endoscopy, ultrasound [26].
Acute kidney injury	• Increase in serum creatinine ≥ 0.3 mg/dL (≥26.5umol/L) from baseline within 48 hours in the patients cirrhosis.[6]
Hypoalbuminemia	• Clinical significant hypoalbuminemia: Serum albumin level < 25g/L [40]
Spontaneous Bacteria Peritonitis	• Ascitic fluid neutrophil count > 250 cells/mm^3 with or without positive culture.[6]
Hyponatremia	• Serum sodium concentration < 130 mmol/L [6]

Appendix 4: Guidelines recommendation for aetiology treatment

Aetiology	First-line medication	Second-line medication
Chronic hepatitis B	Tenofovir disoproxil fumarate: 300mg PO daily<br>Tenofovir alafenamide: 25mg PO daily<br>Entecavir: 0.5mg PO daily<br>[11]	Tenofovir disoproxil fumarate: 300mg PO daily<br>Tenofovir alafenamide: 25mg PO daily<br>Entecavir: 1mg PO daily<br>[11]

Chronic hepatitis C	Sofosbuvir/Velpatasvir: 400mg/100mg tab daily<br>Sofosbuvir/Daclatasvir:<br>Glecaprevir/Pibrentasvir<br>[16]	Sofosbuvir/Velpatasvir/ Voxilaprevir: 400mg/100mg/100mg tab daily<br>[16]
HCC	Systemic therapy<br>Sorafenib; 400mg PO 12 hourly<br>Lenvatinib; < 60 kg: 8mg PO daily, ≥ 60 kg: 12mg PO daily<br>[42]	Systemic therapy<br>Regorafenib: 160mg PO daily<br>Nivolumab; 240mg IV every 2 weeks, 480mg IV every 4 weeks<br>[42]
Alcoholic liver diseases	Alcoholic hepatitis:<br>Prednisolone; 40mg PO daily<br><br>Prevention of alcoholic relapse:<br>Baclofen; 30–60 mg/day<br><br>Alcohol withdrawal:<br>Diazepam; 5–10mg PO 6-8 hourly<br><br>Wernicke encephalopathy:<br>Thiamine; 50-100 mg/day IM/IV	

Appendix 5: Guidelines recommendation for complication treatment

**Complication**	**First-line medication**	**Second-line medication**
Ascites	Spironolactone: 100mg daily up to 400mg<br>Spironolactone +Furosemide: 100mg:40mg daily up to 400mg:160mg daily<br>[22]	Amiloride, Torasemide, Bumetanide, Triamterene, Metolazone, Hydrochlorothiazide [22]
Coagulopathy	Fresh frozen plasma: replacement of coagulation factors<br>Platelet concentrate: if platelet count < 50,000/mm^3<br>Cryoprecipitate: hypofibrinogenemia<br>Vitamin K: 10mg IV daily for 3days<br>[37]	

Esophageal varices	Primary and secondary Prophylaxis of variceal bleeding<br>Non-selective beta blockers<br>Propranolol: 20-40mg PO 12 hourly, maximum 320mg/day in absence of ascites, 160mg/day in ascites<br>Nadolol: 20-40mg PO daily, maximum 160mg/day in absence of ascites, 80mg/day in ascites<br>Carvedilol: 6.25mg PO daily, maximum 12.5mg/day [26]	Vasoactive agents used for acute variceal bleeding:<br>Octreotide<br>Vasopressin<br>Somatosatin<br>Terlipressin<br>[26]
Hepatic encephalopathy	Lactulose<br>Treatment:30-45 mL PO 1-2 hours until at least two soft stools/day, then reduce to 30-45 mL PO 6-8 hourly to produce 2-3 soft stools/day<br>Prophylaxis: 30-45 mL PO, 6-8 hourly, adjusted until to produce 2-3 soft stools/day<br>Rifaximin: 550mg PO 12 hourly [39]	Oral branched chain amino acids (BCAA)<br>L-ornithine L-aspartate (LOLA)<br>Neomycin<br>Metronidazole<br>[39]
Jaundice	There is no specific treatment	
Portal hypertension	There is no specific treatment recommended in the absence of varices [26].	
Acute kidney injury	Withdrawal of nephrotoxic drugs, vasodilators, NSAIDs, diuretics, beta-blockers.<br>Volume expansion with Albumin: 1g/kg for 2 days[6]	
Spontaneous bacterial peritonitis	Cefotaxime: 2g IV 8 hourly x 5 days<br>Ceftriaxone: 1g 12 hourly or 2g daily x 5 days<br>[23]	Ofloxacin: 400mg PO 12 hourly x 8 days<br>Ciprofloxacin: 400mg IV 12 hourly, or 500mg PO 12 hourly x 5 days<br>Levofloxacin: 500mg PO daily, or 750mg IV daily<br>[23]
Hyponatremia	Normal saline in hypovolemic hyponatremia<br>Restriction to 1liter/day in hypervolemic hyponatremia<br>Hypertonic saline<br>[6]	

Appendix 6: Guidelines on safety prescription (https://www.drugsinlivercirrhosis.org)

Drug	Child-Pugh A	Child-Pugh B	Child-Pugh C
Metronidazole	Not yet assessed	Not yet assessed	Not yet assessed
Ciprofloxacin	Not yet assessed	Not yet assessed	Not yet assessed
Ceftriaxone	Not yet assessed	Not yet assessed	Not yet assessed
Metolazone	Not yet assessed	Not yet assessed	Not yet assessed
Glucose	Not yet assessed	Not yet assessed	Not yet assessed
Dextrose 10%	Not yet assessed	Not yet assessed	Not yet assessed
Dextrose 5%	Not yet assessed	Not yet assessed	Not yet assessed
Dextrose5%/saline 0.9%	Not yet assessed	Not yet assessed	Not yet assessed
Vitamin B complex	Not yet assessed	Not yet assessed	Not yet assessed
Multivitamin	Not yet assessed	Not yet assessed	Not yet assessed
Pabrinex	Not yet assessed	Not yet assessed	Not yet assessed
Vitamin K	Not yet assessed	Not yet assessed	Not yet assessed
Hepatovit	Not yet assessed	Not yet assessed	Not yet assessed
Tothema	Not yet assessed	Not yet assessed	Not yet assessed
Vitamin B complex	Not yet assessed	Not yet assessed	Not yet assessed
Multivitamin	Not yet assessed	Not yet assessed	Not yet assessed
Antacids	Not yet assessed	Not yet assessed	Not yet assessed
Folic acid	Not yet assessed	Not yet assessed	Not yet assessed
Ferrous sulphate	Not yet assessed	Not yet assessed	Not yet assessed
Iron dextran	Not yet assessed	Not yet assessed	Not yet assessed
Packed red cells	Not yet assessed	Not yet assessed	Not yet assessed
Whole blood	Not yet assessed	Not yet assessed	Not yet assessed
Albumin	Safe	Safe	Safe
Fresh frozen plasma	Not yet assessed	Not yet assessed	Not yet assessed
Sorafenib	Not yet assessed	Not yet assessed	Not yet assessed
Thiamine	Not yet assessed	Not yet assessed	Not yet assessed
Baclofen	Not yet assessed	Not yet assessed	Not yet assessed
Furosemide	Safe	Safe	Safe
Spironolactone	Safe	Safe	Safe
Furosemide	Safe	Safe	Safe
Paracetamol	Safe	Safe	Safe
Lactulose	Safe	Safe	Safe
Propranolol	Safe	Safe	Safe
Tenofovir	Safe	Safe	Safe
Lamivudine	Safe	Safe	Safe
Tramadol	No adverse effect known	No adverse effects known	No adverse effects known
Morphine	No adverse effect known	No adverse effect known	No adverse effect known
Pethidine	No adverse effect known	No adverse effect known	Unknown
Omeprazole	No adverse effect known	No adverse effect known	Unsafe

Sofosbuvir/Ledipasvir	No adverse effects known	No adverse effects known	No adverse effects known
Ribavirin	No adverse effects known	Adverse effects known	Adverse effects known
Diazepam	Adverse effects known	Adverse effects known	Adverse effects known

Appendix7: Dosing consideration in hepatic impairment (https://www.medscape.com)

Drug class	Child-Pugh A	Child-Pugh B	Child-Pugh C
Metronidazole	No dosage adjustment	No dosage adjustment	Reduce dose by 50%
Ciprofloxacin	No dosage adjustment	No dosage adjustment	No dosage adjustment
Ceftriaxone	No dosage adjustment	Not yet assessed	Not yet assessed
Furosemide	No dosage adjustment	No dosage adjustment	No dosage adjustment
Spironolactone	No dosage adjustment	No dosage adjustment	No dosage adjustment
Metolazone	No dosage adjustment	No dosage adjustment	No dosage adjustment
Glucose	No dosage adjustment	No dosage adjustment	No dosage adjustment
Dextrose 10%	No dosage adjustment	No dosage adjustment	No dosage adjustment
Dextrose 5%	No dosage adjustment	No dosage adjustment	No dosage adjustment
Dextrose/saline	No dosage adjustment	No dosage adjustment	No dosage adjustment
Tramadol	No dosage adjustment	No dosage adjustment	Immediate release, 50mg orally 12 hourly
Paracetamol PO	Use extreme caution; avoid use as much as possible; Limit therapy to short- term use at doses not > 2g/day	Use extreme caution; avoid use as much as possible; Limit therapy to short-term use at doses not > 2g/day	Use extreme caution; avoid use as much as possible; Limit therapy to short- term use at doses not > 2g/day

Morphine	Consider lowest end of dosing range and monitor side effects	Consider lowest end of dosing range and monitor side effects	Consider lowest end of dosing range and monitor side effects
Pethidine	Consider lower limit initial dose initially; increase opioid effect possible in cirrhosis	Consider lower limit initial dose initially; increase opioid effect possible in cirrhosis	Consider lower limit initial dose initially; increase opioid effect possible in cirrhosis
Vitamin B complex	No dosage adjustment	No dosage adjustment	No dosage adjustment
Multivitamin	No dosage adjustment	No dosage adjustment	No dosage adjustment
Pabrinex	No dosage adjustment	No dosage adjustment	No dosage adjustment
Vitamin K	No dosage adjustment	No dosage adjustment	No dosage adjustment
Hepatovit	No dosage adjustment	No dosage adjustment	No dosage adjustment
Omeprazole	Maximum 20mg daily	Maximum 20mg daily	No dosing advice
Antacids	No dosage adjustment	No dosage adjustment	No dosage adjustment
Lactulose	No dosage adjustment	No dosage adjustment	No dosage adjustment
Folic acid	No dosage adjustment	No dosage adjustment	No dosage adjustment
Ferrous sulphate	No dosage adjustment	No dosage adjustment	No dosage adjustment
Iron dextran	No dosage adjustment	No dosage adjustment	No dosage adjustment
Packed red cells	No dosage adjustment	No dosage adjustment	No dosage adjustment
Albumin	No dosage adjustment	No dosage adjustment	No dosage adjustment

Fresh frozen plasma	No dosage adjustment	No dosage adjustment	No dosage adjustment
Propranolol	No dosage adjustment	No dosage adjustment	No dosage adjustment
Tenofovir disproxil fumarate	No dosage adjustment	No dosage adjustment	No dosage adjustment
Lamivudine	No dosage adjustment	No dosage adjustment	No dosage adjustment
Sofosbuvir/ Ledipasvir	No dosage adjustment	No dosage adjustment	No dose adjustment
Ribavirin	No dosage adjustment	No dosage adjustment	No dosage adjustment
Sorafenib	No dosage adjustment	No dosage adjustment	Not studied
Thiamine	No dosage adjustment	No dosage adjustment	No dosage adjustment
Baclofen	No dosage adjustment	No dosage adjustment	No dosage adjustment
Diazepam	Half of maintenance dose	Half of maintenance dose	Half of maintenance dose
